# The prevalence of cardiovascular disease by vascular bed and impact on healthcare costs in a large, real‐world population with type 2 diabetes

**DOI:** 10.1002/edm2.106

**Published:** 2020-01-20

**Authors:** Wayne Weng, Sheldon X. Kong, Rahul Ganguly, Malene Hersloev, Jason Brett, Todd Hobbs, Florian M.M. Baeres

**Affiliations:** ^1^ Novo Nordisk Inc Plainsboro NJ USA; ^2^ Novo Nordisk A/S Søborg Denmark

**Keywords:** atherosclerotic cardiovascular disease, cerebrovascular disorders, coronary disease, epidemiology, peripheral vascular diseases, type 2 diabetes mellitus, vascular diseases

## Abstract

**Introduction:**

The purpose of this study was to assess prevalence of atherosclerotic cardiovascular disease (ASCVD) according to number of affected vascular beds and the impact on healthcare utilization and costs in persons with type 2 diabetes mellitus (type 2 DM) and established ASCVD.

**Methods:**

In this retrospective, cross‐sectional analysis, adults with type 2 DM and ASCVD in a large US administrative claims database were categorized by number of ASCVD‐affected vascular beds (brain, heart, peripheral vasculature). Annual healthcare utilization and costs for 2015 were determined, including subgroup analyses by age group (18‐44, 45‐64, ≥65 years).

**Results:**

Among 539 089 individuals with type 2 DM and ASCVD, 47.0% had ASCVD affecting >1 vascular bed. The most prevalent ASCVD diagnoses were acute coronary syndrome (26.6%), peripheral arterial disease (24.5%) and stroke (18.6%). Mean annual total healthcare costs per person increased with increasing number of vascular beds, from 1 ($17 741) to 2 ($25 877) to 3 ($33 412). A similar pattern of increased healthcare utilization with increasing number of vascular beds was observed. Among individuals with 1 affected vascular bed, mean total healthcare costs per person were comparable across age subgroups; however, if >1 vascular bed was affected, the mean total healthcare costs were highest in the youngest age cohort.

**Conclusions:**

These real‐world data showed that almost half of individuals with type 2 DM and ASCVD had ASCVD affecting >1 vascular bed. A higher number of affected vascular beds were associated with higher mean total healthcare costs and utilization, with a disproportionate increase noted in younger relative to older people.

## Graphical abstract

There are limited data regarding the healthcare cost impact of ASCVD based on the number of affected vascular beds. This analysis, using claims data from 539 089 individuals with type 2 DM and ASCVD, found that 47.0% (n = 253 548) had ASCVD affecting >1 vascular bed, and a higher number of affected vascular beds were associated with higher mean total healthcare costs. These findings underscore the economic burden of ASCVD in people with type 2 DM, particularly among those with multiple affected vascular beds.

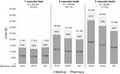

## 1 INTRODUCTION

Type 2 diabetes mellitus (type 2 DM) is a highly prevalent disease and associated with a large economic burden for individuals and healthcare systems.^1^, ^2^ In the United States (US), the annual cost of diabetes is approximately $327 billion (2017 estimate).^3^ Atherosclerotic cardiovascular disease (ASCVD) is a major cause of morbidity and mortality in type 2 DM^4^, ^5^ and contributes to the economic burden of type 2 DM.^6^ ASCVD affecting multiple vascular beds (ie, brain, heart, peripheral vascular) has been associated with higher vascular event rates than single‐bed disease.^7^ In addition, people with diabetes have been reported to have increased plaque burden and more diffuse ASCVD, possibly due to mechanisms inherent to diabetes such as hyperglycaemia‐induced increases in oxidative stress.^8^, ^9^

There are limited data regarding the healthcare cost impact of ASCVD based on the number of affected vascular beds, particularly in people with type 2 DM. In the large, international REduction of Atherothrombosis for Continued Health (REACH) Registry, data for 23 974 participants in the United States revealed a significant trend of higher hospitalization costs as the number of affected vascular beds increased.^10^ To our knowledge, there have been no studies of this kind in a population with diabetes.

We have reported previously, using data from a large US claims database, that the presence of ASCVD was associated with significantly higher healthcare costs in persons with type 2 DM.^11^ The current analysis, using the same real‐world claims database, focused on adults with type 2 DM and established ASCVD and assessed the prevalence of ASCVD according to the number of affected vascular beds, as well as the impact on healthcare utilization and costs.

## 2 PARTICIPANTS AND METHODS

### 2.1 Data source and study population

This was a retrospective, cross‐sectional analysis using data from a large, nationwide US administrative claims database (IBM^®^ Family of MarketScan^®^ Research Databases, formerly Truven Health Analytics MarketScan Databases) using 2015 data. The MarketScan database contains de‐identified, individual healthcare claims data from all states in the United States and is fully compliant with the Health Insurance Portability and Accountability Act of 1996.

The claims database population used in the current study has been described previously.^11^, ^12^ Briefly, eligible individuals were aged ≥18 years on 1 January 2015 and had an established diagnosis of type 2 DM before 1 January 2015, defined as ≥2 diagnoses for type 2 DM, based on international classification of diseases, ninth revision (ICD‐9) codes of 250.×0 or 250.×2 or ICD‐10 codes of E11.xx or ≥1 type 2 DM diagnosis with ≥1 oral antidiabetes drug claim, and no more than 1 type 1 diabetes diagnosis according to ICD‐9 (250.×1, 250.×3) or ICD‐10 (E10.×) codes. Continuous health plan enrolment with an insurance plan containing both medical and pharmacy benefits between 1 January 2014 and 31 December 2015 was required. The baseline period was defined as 1 January 2014 to 31 December 2014, and the study period was defined as 1 January 2015 to 31 December 2015.

This analysis included adults with type 2 DM and at least one ICD‐9 diagnosis code for ASCVD prior to 1 January 2015 (see Supplemental Table 1 for listing of codes) corresponding to the ADA 2017 Standards of Medical Care definition of ASCVD: acute coronary syndrome (ACS), history of myocardial infarction (MI), stable or unstable angina pectoris, peripheral arterial disease (PAD) presumed to be of atherosclerotic origin, stroke, transient ischaemic attack (TIA) and coronary or other arterial revascularization.^13^

**Table 1:** Demographic characteristics of a real‐world 2015 population with type 2 DM and ASCVD^a^ (N = 543,938)

Variable	All	Number of vascular beds affected		
		1	2	3
Individuals, n (%)	539 089 (100.0)	285 541 (53.0)	156 856 (29.1)	96 692 (17.9)
Age, years, mean (SD)	66.6 (12.2)	63.0 (11.6)	68.6 (11.7)	73.8 (11.0)
Range	18‐108	18‐104	19‐106	20‐108
Age category, n (%)				
18‐44 years	16 565 (3.1)	13 402 (4.7)	2639 (1.7)	524 (0.5)
45‐64 years	251 713 (46.7)	165 870 (58.1)	63 545 (40.5)	22 298 (23.1)
≥65 years	270 811 (50.2)	106 269 (37.2)	90 672 (57.8)	73 870 (76.4)
Sex, n (%)				
Women	253 400 (47.0)	137 049 (48.0)	72 852 (46.5)	43 499 (45.0)
Men	285 689 (53.0)	148 492 (52.0)	84 004 (53.6)	53 193 (55.0)
Region of United States, n (%)				
North Central	171 406 (31.8)	77 270 (27.1)	52 294 (33.3)	41 842 (43.3)
Northeast	112 508 (20.9)	60 656 (21.2)	33 785 (21.5)	18 067 (18.7)
South	205 969 (38.2)	118 576 (41.5)	57 438 (36.6)	29 955 (31.0)
West	48 144 (8.9)	28 379 (9.9)	13 041 (8.3)	6724 (7.0)
Unknown	1062 (0.2)	660 (0.2)	298 (0.2)	104 (0.1)
Insurance, n (%)				
Commercial	271 160 (50.3)	181 877 (63.7)	66 768 (42.6)	22 515 (23.3)
Medicare	267 929 (49.7)	103 664 (36.3)	90 088 (57.4)	74 177 (76.7)

a As defined by ADA 2017 guidelines.

Abbreviation: ASCVD, atherosclerotic cardiovascular disease; DM, diabetes mellitus.

Each ASVCD diagnosis was classified as one of three vascular beds: brain (stroke, TIA), heart (ACS, angina, MI) or peripheral vasculature (PAD). Patients were then categorized by the number of vascular beds affected by their ASCVD diagnoses (ie, 1, 2 or 3 vascular beds).

### 2.2 Variables of interest

Population demographics were determined as of 1 January 2015 and included age, sex, geographic region and insurance type. Study end‐points included all‐cause annual healthcare costs per person for 2015 (medical, pharmacy and total costs) and healthcare utilization (inpatient, emergency room [ER] and outpatient visits) determined for each person and compared between groups with 1, 2 or 3 affected vascular beds.

### 2.3 Data analysis

This was a descriptive analysis. Population characteristics were measured using counts with percentages for the categorical variables and means with standard deviation for continuous variables. Subgroup analyses were conducted for 3 age categories: 18‐44, 45‐64 and ≥65 years.

## 3 RESULTS

### 3.1 Study population

**Figure 1:**
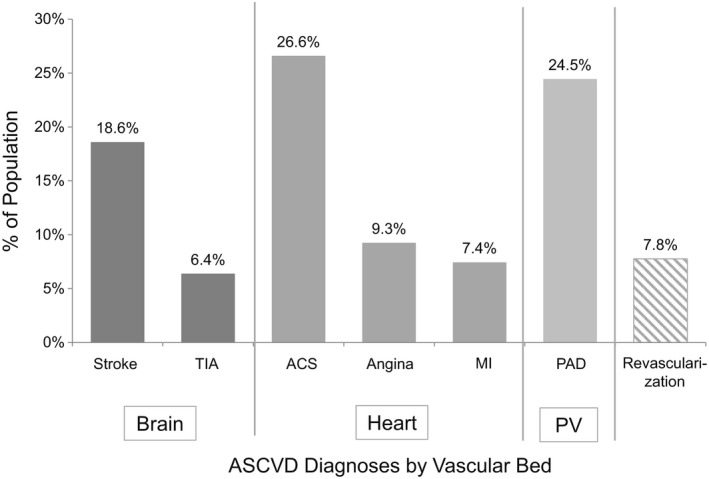
ASCVD^a^ prevalence within vascular bed categories in a real‐world population of 539 089 adults with type 2 DM. Note: Revascularization was included in the 2017 ADA guidelines definition of ASCVD but not easily categorized by vascular bed. ^a^ASCVD as defined by 2017 ADA guidelines (ADA, 2017). ACS, acute coronary syndrome; ASCVD, atherosclerotic cardiovascular disease; MI, myocardial infarction; PAD, peripheral arterial disease; PV, peripheral vasculature; type 2 DM, type 2 diabetes mellitus; TIA, transient ischaemic attack

Of 1 202 596 individuals with type 2 DM who met study eligibility criteria, 539 089 (44.8%) had one or more ASCVD‐related diagnoses and were included in the analysis (Table 1). The study cohort had a mean age of 66.6 years and was comprised of fairly similar proportions of men (47.0%) and women (53.0%). Most of the people were in the age groups of ≥65 years (50.2%) or 45‐64 years (46.7%), with a small percentage between the ages of 18 and 44 (3.1%). Geographically, most people were from the US South (38.2%) and North Central (31.8%) regions. Overall, half of the population had commercial insurance, and half had Medicare. The most common ASCVD diagnoses were ACS (26.6%), PAD (24.5%) and stroke (18.6%) (Figure 1).

### 3.2 Affected vascular beds

ASCVD affecting only 1 vascular bed was most prevalent (53.0% of people), while 29.1% and 17.9% of people had ASCVD affecting 2 or 3 vascular beds, respectively (Table 1). Mean age increased with increasing number of affected vascular beds, ranging from 63.0 years in the 1 affected vascular bed subgroup to 73.8 years in the 3 affected vascular bed subgroup. The gender distribution stayed relatively consistent regardless of the number of affected vascular beds.

Table 2 provides a breakdown of affected vascular beds for the entire study population and by age category. For the 18‐44 age subgroup, a majority (80.9%) of individuals had 1 affected vascular bed, and few (3.2%) had 3 affected vascular beds. In the 45‐64 age subgroup, 65.9% had 1 affected vascular bed and 8.9% had 3 affected vascular beds. This distribution was markedly different in the ≥65 years age subgroup, in which 39.2% had 1 affected vascular bed and 27.3% had 3 affected vascular beds. For the population overall, the heart was the most commonly affected vascular bed (65.3% of people), followed by PAD (54.6%) and brain (45.1%); this pattern was similar within each age category except for the 18‐44 subgroup in which PAD was the most prevalent vascular bed affected (49.2%), followed by heart (44.1%) and brain (29.0%).

**Table 2:** Prevalence of ASCVD by number of affected vascular beds and age category

Vascular Beds Affected	Age category, n (% within age category)			
	All N = 539 089	18‐44 n = 16 565	45‐64 n = 251 713	≥65 n = 270 811
1 Vascular Bed	285 541 (53.0)	13 402 (80.9)	165 870 (65.9)	106 269 (39.2)
Heart only	139 204 (25.8)	5129 (31.0)	83 235 (33.1)	50 840 (18.8)
PAD only	89 760 (16.7)	5591 (33.8)	52 489 (20.9)	31 680 (11.7)
Brain only	56 577 (10.5)	2682 (16.2)	30 146 (12.0)	23 749 (8.8)
2 vascular beds	156 856 (29.1)	2639 (15.9)	63 545 (25.2)	90 672 (33.5)
Heart + PAD	67 122 (12.5)	1046 (6.3)	28 542 (11.3)	37 534 (13.9)
Heart + Brain	49 216 (9.1)	601 (3.6)	18 823 (7.5)	29 792 (11.0)
PAD + Brain	40 518 (7.5)	992 (6.0)	16 180 (6.4)	23 346 (8.6)
3 vascular beds^a^	96 692 (17.9)	524 (3.2)	22 298 (8.9)	73 870 (27.3)
Any heart	352 234 (65.3)	7300 (44.1)	152 898 (60.7)	192 036 (70.9)
Any PAD	294 092 (54.6)	8153 (49.2)	119 509 (47.5)	166 430 (61.5)
Any brain	243 003 (45.1)	4799 (29.0)	87 447 (34.7)	150 757 (55.7)

Note

Because of rounding, some categories may differ by 0.1 from the total for that category.

Abbreviation: ASCVD, atherosclerotic cardiovascular disease; PAD, peripheral arterial disease.

a Heart + PAD + Brain.

### 3.3 Healthcare costs and utilization

Mean total healthcare costs per person increased with increasing number of affected vascular beds, ranging from $17 741 in people with 1 affected vascular bed to $33 412 in those with 3 affected vascular beds, representing an increase of 88.3% (Table 3). Cost differences were impacted primarily by increasing medical costs. Relative to people with 1 affected vascular bed only, the mean total annual medical costs per person were 59.7% and 117.4% higher for those with 2 or 3 affected vascular beds, respectively. Pharmacy costs were also higher but to a much smaller degree among people with 2 (9.4% higher) or 3 affected vascular beds (11.8% higher).

**Table 3:** Per person annual (2015) healthcare costs and utilization by number of vascular beds affected

	Number of affected vascular beds				
	1 (N = 285 541)	2 (N = 156 856)	% diff^a^	3 (N = 96 692)	% diff^a^
Total costs, US$, mean	$17 741	$25 877	+45.9	$33 412	+88.3
Medical^b^	$12 855	$20 529	+59.7	$27 947	+117.4
Pharmacy	$4887	$5348	+9.4	$5464	+11.8
Utilization, number of visits, mean ± SD					
Outpatient^c^	18.15	24.12	+32.9	31.57	+73.9
Inpatient	0.13	0.24	+84.6	0.38	+192.3
ER, inpatient^d^	0.03	0.06	+100.0	0.10	+233.3
ER, outpatient^e^	0.41	0.62	+51.2	0.98	+139.0

Note

Because of rounding, total cost may differ from the sum of Medical + Pharmacy costs by $1

ER, emergency room.

a Relative to people with 1 affected vascular bed

b Medical represents nonpharmacy healthcare costs, including outpatient, inpatient and ER visits

c Includes outpatient office/facility and laboratory visits

d ER visit resulting in inpatient admission

e ER visit not resulting in inpatient admission

Mean healthcare utilization was markedly higher in people with higher numbers of affected vascular beds (Table 3). The greatest differential in utilization was observed for ER inpatient visits which were 100.0% and 233.3% higher among individuals with 2 or 3 affected vascular beds, respectively, relative to those with 1 affected vascular bed. Inpatient admissions were increased to almost the same extent (84.6% and 192.3%, respectively), as well as ER outpatient visits (51.2% and 139.0%, respectively). Comparatively, annual outpatient visits were impacted on a smaller scale, yet increased from 18.15 visits per person per year in people with 1 affected vascular bed to 24.12 (32.9% increase) and 31.57 (73.9% increase) visits per person per year in those with 2 or 3 affected vascular beds, respectively.

### 3.4 Subgroup analysis by age category

**Figure 2:**
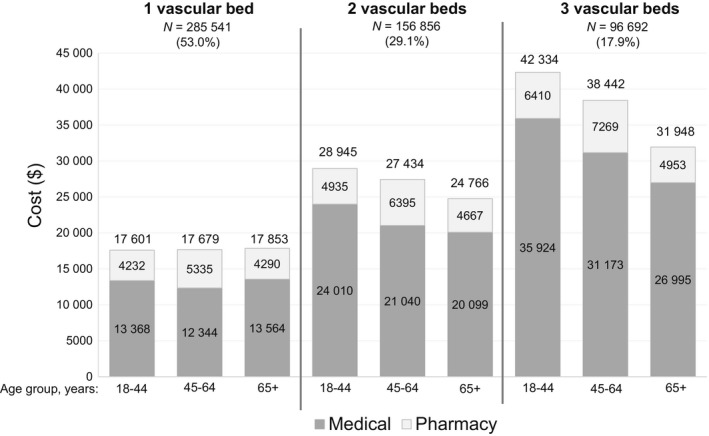
Mean annual total healthcare costs per person (2015), including medical^a^ and pharmacy cost contributions, by number of vascular beds affected and age category. ^a^Medical represents nonpharmacy healthcare costs, including outpatient, inpatient and emergency room visits

Mean healthcare cost comparisons across age groups (18‐44, 45‐64 and ≥65) revealed different patterns as the number of affected vascular beds increased (Figure 2). Among individuals with ASCVD affecting only 1 vascular bed, mean total healthcare costs per person were similar across the 3 age subgroups; however, if 2 or 3 vascular beds were affected, the mean total healthcare cost was negatively associated with age (ie, costs were higher in younger age groups).

**Figure 3:**
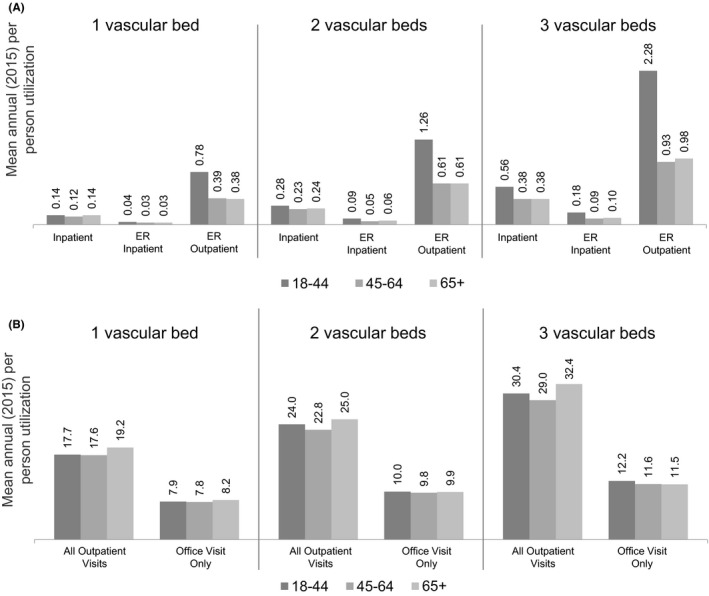
Mean annual (2015) per person healthcare utilization by number of vascular beds and age category. (**A) Inpatient and emergency visits,** emergency room (ER) inpatient, emergency department visit resulting in inpatient admission, ER outpatient, emergency department visit not resulting in inpatient admission. (**B) Outpatient visits,** “All outpatient” visits include outpatient physician office visits as well as any other outpatient facility visits, laboratory testing, etc

With regard to healthcare utilization by age group and number of affected vascular beds, mean annual inpatient and outpatient utilization rates were generally similar between age groups within the same vascular bed category (Figure 3). The most notable difference between age groups was observed for ER outpatient visit frequencies (ER visits not resulting in inpatient admission); regardless of number of affected vascular beds, the youngest age group had approximately double the number of mean annual ER outpatient visits compared with the other age groups in the same vascular bed category.

## 4 DISCUSSION

This real‐world cross‐sectional analysis examined claims data from a large national cohort of adults with type 2 DM and ASCVD. More than 1 vascular bed was affected in almost half (47.0%) of the study cohort, with ASCVD diagnoses affecting the heart being most prevalent. In addition, individuals with 3 affected vascular beds had a mean age of approximately 11 years greater than those with 1 affected vascular bed.

Mean total healthcare costs per person increased with increasing number of affected vascular beds and were nearly double for people with 3 affected vascular beds compared with those with 1 affected vascular bed. Of note, while age did not appear to influence mean healthcare costs among individuals with 1 affected vascular bed, the finding of relatively higher mean healthcare costs in younger age groups among people with >1 affected vascular bed was unexpected and without explanation. The most notable discrepancy in annual utilization between age groups was the higher rates of outpatient ER utilization among younger people, and this may have been a contributing factor.

The presence of ASCVD in adults with type 2 DM is well known to exert an additive cost burden. We have previously used MarketScan claims data to compare healthcare utilization and costs among people with type 2 DM with or without ASCVD (regardless of vascular bed categories) and found that the mean total healthcare cost in the ASCVD group (approximately $23 000) was more than twice that in the group without ASCVD (approximately $10 000).^11^ Similarly, another electronic medical records and claims database study found that the average total healthcare costs per person per month were almost double in people with type 2 DM with cardiovascular disease (CVD; defined as diagnosis of stroke, TIA, MI, unstable angina or coronary revascularization), as compared with those without CVD.^14^ The data from the current analysis extend these previous findings by showing that people with a higher number of affected vascular beds had higher mean healthcare costs, suggesting that more extensive ASCVD may be associated with higher economic burden.

Our finding that almost half of adults with type 2 DM and ASCVD had more than 1 affected vascular bed is consistent with other published reports describing people with diabetes as having extensive ASCVD.^8^, ^9^ Considering that involvement of multiple vascular beds has been associated with a higher rate of vascular events,^7^ we suspect that this could contribute to higher healthcare costs associated with ASCVD in adults with type 2 DM, although our study did not evaluate ASCVD event rates and this is speculative. Regardless, cost‐effective treatment strategies that address both type 2 DM and ASCVD are clearly needed.

This analysis has certain limitations. Identification of type 2 DM and ASCVD was based on ICD‐9/‐10 codes which may be impacted by provider coding practices and are subject to coding error. Diagnosis of type 2 DM required corroboration beyond a single claim, although ASCVD diagnoses were included even if only a single claim was present. Yet, the sheer size of the database would be expected to minimize any potential impact of occasional coding errors. In addition, it is possible that other personal and disease variables (eg, chronic kidney disease, including end‐stage renal disease) influenced healthcare utilization and costs, beyond the number of vascular beds affected by ASCVD, and this study was not designed to evaluate the contribution of other factors. In addition, the sample size of the 18‐44 age cohort was very small compared to the other two older cohorts, and this makes the findings on the 18‐44 cohort less robust and should be interpreted with caution. Finally, while the sample was geographically representative within the US, the findings may not be relevant to non‐US countries.

In conclusion, this descriptive analysis of a large, nationwide, real‐world sample of over a half‐million adults with type 2 DM and ASCVD found that almost half of such individuals had ASCVD in multiple vascular beds. Further, mean total healthcare costs and utilization increased with increasing number of affected vascular beds. Despite the limitations of the study, these findings provide real‐world insight regarding the clinical and economic burden of multivascular bed ASCVD in people with type 2 DM, which we hope stimulates additional research into this phenomenon.

## CONFLICTS OF INTEREST

R. Ganguly, M. Hersloev, J. Brett and T. Hobbs are current employees of Novo Nordisk Inc, the funding body for this study. F. M. M. Baeres is a current employee of Novo Nordisk A/S. W. Weng and S. X. Kong were employees of Novo Nordisk, Inc, during study conduct.

## Data Availability

The data that support the findings of this study are available from IBM/Truven. Restrictions apply to the availability of these data, which were used under licence for this study. Data are available Wayne Weng with the permission of IBM/Truven.
